# Copper(II), Nickel(II) and Zinc(II) Complexes of Peptide Fragments of Tau Protein

**DOI:** 10.3390/molecules29102171

**Published:** 2024-05-07

**Authors:** Zsuzsa Kastal, Adrienn Balabán, Szilvia Vida, Csilla Kállay, Lajos Nagy, Katalin Várnagy, Imre Sóvágó

**Affiliations:** 1 Department of Inorganic and Analytical Chemistry, University of Debrecen, Egyetem tér 1, H-4032 Debrecen, Hungary; kastal.zsuzsa@science.unideb.hu (Z.K.); kallay.csilla@science.unideb.hu (C.K.); varnagy.katalin@science.unideb.hu (K.V.); 2 Department of Applied Chemistry, University of Debrecen, Egyetem tér 1, H-4032 Debrecen, Hungary; nagy.lajos@science.unideb.hu

**Keywords:** tau protein, histidine-containing fragments, copper(II) complex, zinc(II) complex, nickel(II) complex, copper(II) catalyzed oxidation, potentiometry, UV-Vis spectroscopy, CD spectroscopy

## Abstract

Copper(II), nickel(II) and zinc(II) complexes of various peptide fragments of tau protein were studied by potentiometric and spectroscopic techniques. All peptides contained one histidyl residue and represented the sequences of tau(91–97) (Ac-AQPHTEI-NH_2_), tau(385–390) (Ac-KTDHGA-NH_2_) and tau(404–409) (Ac-SPRHLS-NH_2_). Imidazole-N donors of histidine were the primary metal binding sites for all peptides and all metal ions, but in the case of copper(II) and nickel(II), the deprotonated amide groups were also involved in metal binding by increasing pH. The most stable complexes were formed with copper(II) ions, but the presence of prolyl residues resulted in significant changes in the thermodynamic stability and speciation of the systems. It was also demonstrated that nickel(II) and especially zinc(II) complexes have relatively low thermodynamic stability with these peptides. The copper(II)-catalyzed oxidation of the peptides was also studied. In the presence of H_2_O_2_, the fragmentation of peptides was detected in all cases. In the simultaneous presence of H_2_O_2_ and ascorbic acid, the fragmentation of the peptide is less preferred, and the formation of 2-oxo-histidine also occurs.

## 1. Introduction

Neurological disorders are generally linked to abnormal conformational changes in various proteins. Tau protein is one of these molecules. It contains 441 amino acids, and 12 of them are histidine, which is the most common metal binding site in various proteins. As a consequence, tau protein is a potentially effective metal-binding molecule, but its coordination behavior is much less studied as compared to other biomolecules related to neurodegeneration. In the case of amyloid-β and prion proteins, a huge number of studies are available on the metal-binding ability of both the whole protein and various peptide fragments. The most important results obtained in these fields are summarized in various reviews and strongly support the high metal ion affinity of these substances [1,2,3,4,5]. In a more recent review, we compared the copper(II)- and zinc(II)-binding ability of various peptide fragments of amyloid-β, prion and tau proteins [6]. This comparison revealed that imidazole-N donors are the primary metal binding sites for all peptides and for both metal ions. The stoichiometry and coordination modes of the different peptides, however, can be significantly different. The number and location of histidyl residues in the sequence and the presence of other coordinating side chains are the governing factors during complex formation. In the case of the prion protein, the well-separated histidyl residues and the absence of other strongly coordinating side chains result in relatively simple complex formation processes, and all histidyl moieties can be independent metal binding sites when copper is abundant, while the formation of macrochelates is favored under low copper occupancy. Amyloid-β is richer in possible coordination sites involving the terminal amino and carboxylate groups of aspartyl and glutamyl residues, and the histidine can be in either a vicinal or distant position. Thus, the coordination chemistry of amyloid-β is more complex than that of prion protein fragments. Amyloid-β can effectively bind both copper(II) and zinc(II) ions, while copper(II) binding is much more favored for prion peptides [7,8,9]. The amino acid sequence of tau protein also includes both distant and vicinal histidyl residues, and peptides are rich in the polar side chains of aspartyl, glutamyl, seryl and threonyl residues. It is also important to note that tau protein also contains cysteinyl residues, which can be potential binding sites for both copper(II) and zinc(II) ions and can contribute to tau aggregation [10,11,12]. Most previous studies have focused on the R3 and R4 regions of the protein, which are considered the microtubule-binding domains of the protein [13,14,15], but the possible biological role of the N-terminal region of the protein has also been investigated [16,17].

According to a literature review and clinical observations, oxidative stress is suggested as the dominant factor for the pathogenesis of Alzheimer’s disease [18,19]. The binding of certain metal ions, such as Cu(II), to amyloid-β can promote the production of reactive oxygen species. This may contribute to oxidative damage to both the amyloid-β peptide itself and the surrounding molecules [20]. A similar process may occur with tau protein. However, the copper(II) ion-catalyzed oxidation of tau protein is especially poorly characterized. Nevertheless, the comparison of oxidation reactions with those of other neuronal proteins, such as the prion protein or amyloid-β peptides, is summarized in our recent review [6]. But systematic studies are not available in the literature in this field.

In the last few years, we have started to perform systematic studies on the metal complexes of various regions of tau protein for a better understanding and comparison of the metal-binding ability of the various peptide fragments. Tau(9–16) and tau(26–33) containing His14 and His32 residues, respectively, and their mutants were involved in the first study, and the results revealed a slight preference for copper(II) binding at His14 over His32 in slightly acidic conditions. The stability order, however, changed with the physiological pH range when amide-bonded species were formed [21]. Interestingly, this preference persisted even in the presence of the fragment tau(326–333), although this peptide contains two vicinal histidyl residues (His329 and His330) [22]. On the contrary, His14 and His32 have a low zinc(II)-binding affinity, and the Zn(II)-N_im_-coordinated species cannot compete with the hydrolytic reactions of the metal ions. The amide-coordinated zinc(II) complexes of tau(326–333), however, predominate in slightly alkaline samples [23]. Now, in this paper, we report the results of combined potentiometric and spectroscopic studies for the copper(II), zinc(II) and nickel(II) complexes of three other peptide fragments of tau protein: tau(91–97) (Ac-AQPHTEI-NH_2_), tau(385–390) (Ac-KTDHGA-NH_2_) and tau(404–409) (Ac-SPRHLS-NH_2_). All these peptides contain only one histidyl residue (H95, H388 and H407, respectively), but they are present in different chemical environments.

## 2. Results and Discussion

### 2.1. The Acid–Base Properties of the Peptides

The protonation constants of the peptides were determined by potentiometric titrations using the procedure described in the experimental section. Since all peptides are in N- and C-terminally protected forms, in addition to the histidyl sites, only the side chain carboxylate groups of aspartyl or glutamyl and the amino groups of lysyl residues can take part in the protonation equilibria of these molecules. The corresponding p*K* values are listed in Table 1, and data published earlier for tau(9–16) and tau(26–33) are also included in this table for comparison.

It is clear from Table 1 that the number of protonation sites is very different among the peptide fragments, but with a comparison with previous literature data, these values can be easily assigned to the preferred protonation sites. The p*K* values of imidazole-N donors in the absence of other side chains are generally around 6–6.3 [5], and the value measured for tau(404–409) is in good agreement with this expectation. In the case of tau(91–97) and tau(385–390), the imidazole p*K* values are slightly higher because of the presence of the acidic aspartyl or glutamyl residues. The carboxylic groups of these residues can form weak hydrogen bonds with the imidazole-N atom and slightly hinder its deprotonation. The high p*K* value for the lysyl ammonium group is also characteristic of peptides.

### 2.2. Copper(II) Complexes of the Peptides

**Figure 1:**
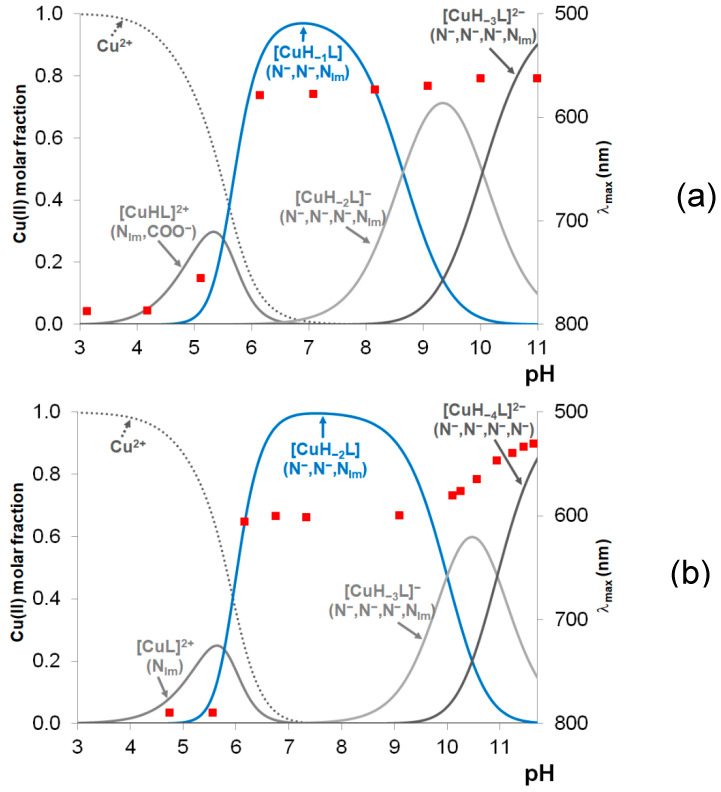
Metal ion speciation in the copper(II)-tau(385–390) (**a**) and copper(II)-tau(404–409) (**b**) systems (c(L) = c(M) = 1.2 mM) together with the change in absorption maxima (marked with red squares).

The stability constants of the copper(II) complexes were determined by potentiometric titrations, and the data are included in Table 2. All peptides have only one effective anchoring site for metal ion coordination, and only mononuclear complexes are formed in these systems. The formation of bis(ligand) complexes cannot be ruled out, but the bulky side chains of the ligands and the preferred amide coordination suppress the formation of this type of complex. Spectroscopic data strongly suggest that metal ion coordination starts with Cu(II)-N_im_ binding in acidic media. The stability constant of the Cu(II)-1N_im_-bonded species is generally around 4 log units [24], and similar values can be found in Table 2. The highest value was obtained for the hexapeptide tau(91–97), and it can be easily explained by the presence of a glutamyl carboxylate group in the vicinity of the histidyl residue. The speciation curves for the copper(II)-tau(385–390) and copper(II)-tau(404–409) systems are plotted in Figure 1a,b, respectively. It is clear from these Figures that the simple Cu(II)-N_im_-coordinated complexes ([CuL] or [CuHL]) are present in low concentrations and exist only in acidic media.

**Figure A1:**
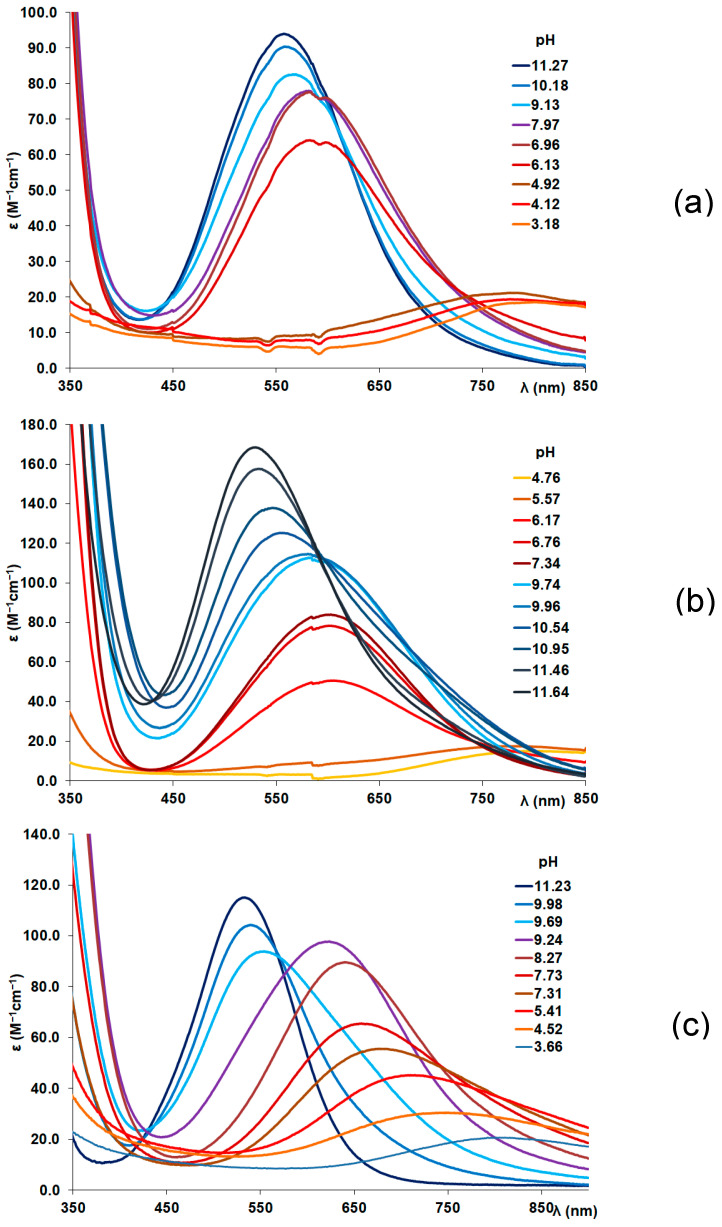
pH-dependent UV-Vis spectra of Cu(II)-tau(385–390) at 1:1 ratio, c(L) = 1.2 mM (**a**); Cu(II)-tau(404–409) at 1:1 ratio, c(L) = 1.2 mM (**b**); and Cu(II)-tau(91–97) at 1:2 ratio, c(L) = 0.9 mM (**c**).

The visible absorption maxima of the solutions are also plotted in Figure 1a,b, and it is evident that the formation of the (N^−^,N^−^,N_im_)-coordinated species ([CuH_−2_L] or [CuH_−2_LH] = [CuH_−1_L]) from the 1N_im_-coordinated one is accompanied by a significant blue shift of the absorption maxima (from 780 to 580–600 nm; see Table 3 and Figure A1 in Appendix A).

In agreement with previous findings obtained for copper(II) complexes of peptides including internal histidyl residues, these spectral changes can be explained by the deprotonation and metal ion coordination of the amide bonds of peptides preceding the histidyl residue. It is also evident that the deprotonation and metal ion coordination of the first two amide functions take place in a cooperative manner, resulting in the formation of [CuH_−2_L] or [CuH_−2_LH]. This is also a common feature of all N-terminally protected peptides containing histidine [5]. In the case of the tau(385–390) peptide, the deprotonation of the third amide nitrogen occurs above pH 8.5 ([CuH_−2_L]), followed by the deprotonation of the lysine side chain ammonium group ([CuH_−3_L]). The absorption maximum characteristic of the (N^−^,N^−^,N^−^,N_im_)-coordinated complex appears at a wavelength about 30 nm higher than that of other protected 1-His-containing peptides (e.g., tau(9–16)). For [CuH_−3_L] of the tau(26–33) peptide, which also contains a -TXH- sequence, a similar λ_max_ value is characteristic (Table 3). In both cases, weak axial coordination of the Thr-OH group may result in a slight red shift of the absorption maximum.

**Figure 2:**
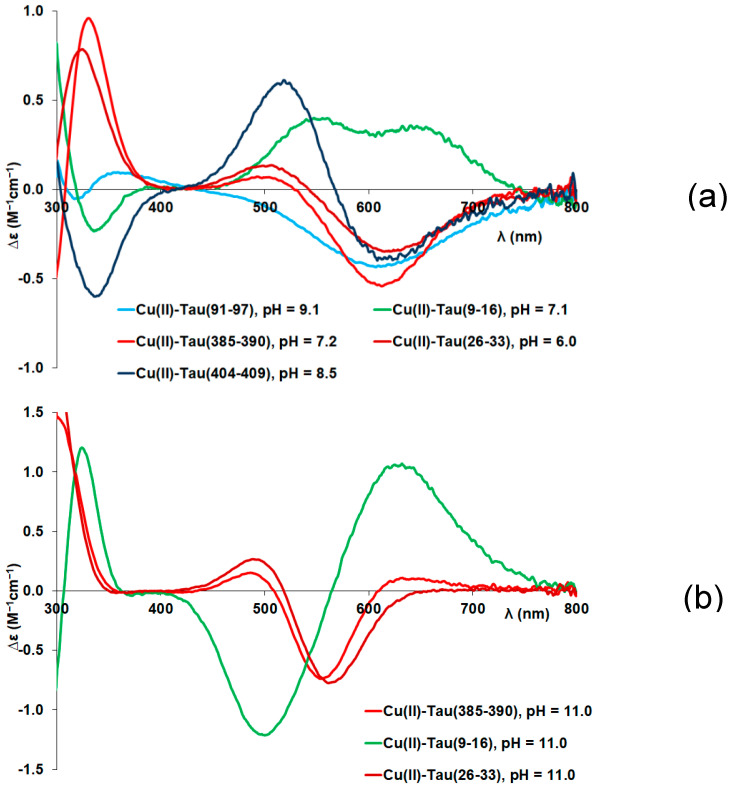
Circular dichroism (CD) spectra of (N^−^,N^−^,N_im_)-coordinated [CuH_−2_L] (3N) complexes (**a**) and (N^−^,N^−^,N^−^,N_im_)-coordinated [CuH_−3_L] (4N) complexes (**b**).

**Figure A2:**
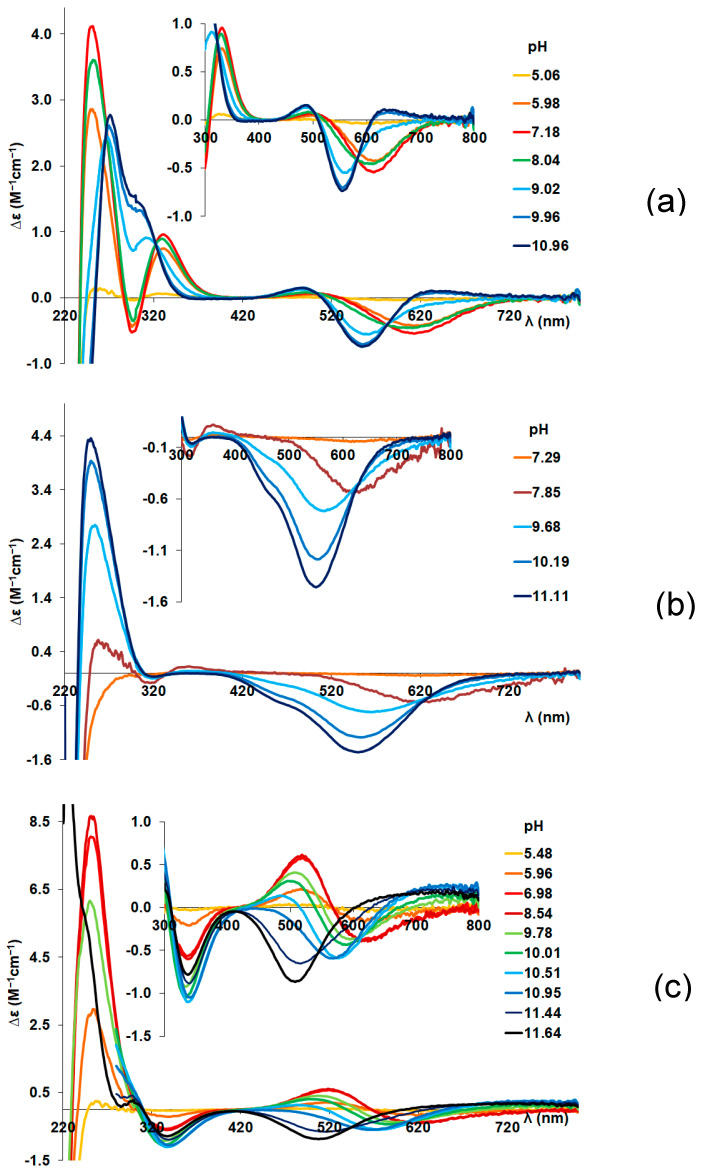
pH-dependent CD spectra of the Cu(II)-tau(385–390) at 1:1 ratio, c(L) = 1.2 mM (**a**); Cu(II)-tau(404–409) at 1:1 ratio, c(L) = 1.2 mM (**b**); and Cu(II)-tau(91–97) at 1:2 ratio, c(L) = 0.9 mM (**c**).

Circular dichroism (CD) spectra provide further support for the above-mentioned coordination modes. Measurable CD spectra can only be detected above pH 5 in parallel with the formation of amide-coordinated complexes (Figure A2 in Appendix A). These spectra are plotted in Figure 2a for the (N^−^,N^−^,N_im_)-coordinated [CuH_−2_L] or [CuH_−2_LH] (3N complexes) and Figure 2b for the (N^−^,N^−^,N^−^,N_im_)-coordinated [CuH_−3_L] species (4N complexes). The corresponding spectra of the complexes formed with the N-terminal fragments (tau(9–16) and tau(26–33)) are also shown for comparison.

It is evident that the 3N- and 4N-coordinated copper(II) complexes have significantly different spectral characteristics, suggesting that the coordination of the first two amide-N atoms occurs cooperatively in the pH range 5–7, while the last deprotonation process is well separated and shifted to a slightly alkaline media (pH ~ 8–10). It is also obvious from the comparison of CD spectra that the spectrum of peptide tau(9–16) differs from the others. Similar differences have already been observed for other peptides containing threonyl or seryl residues preceding the anchoring histidyl residue by two amino acids in the sequence. This difference was explained by the effect of the presence of the hydroxylic OH groups of the threonine and serine amino acids [25,26,27].

**Figure A3:**
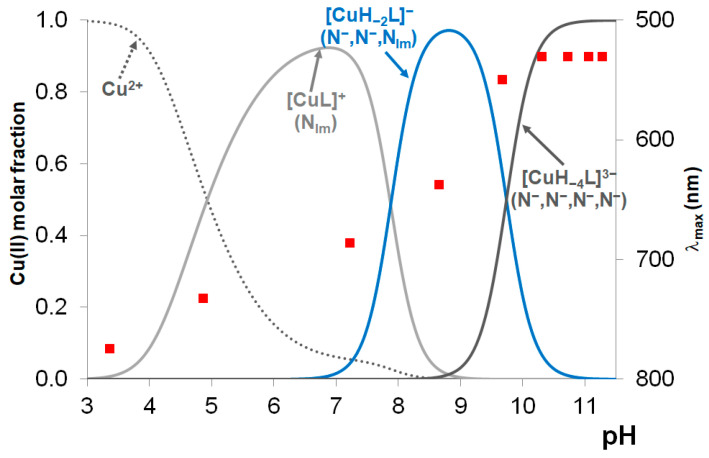
Metal ion speciation in the copper(II)-tau(91–97) system (c(L) = c(M) = 0.9 mM) together with the change in absorption maxima (marked with red squares).

In addition to the above-mentioned effect of threonyl residues, complex formation by peptides containing proline also differs from the others. This difference is reflected in the values of stability constants and in the speciation curves and characteristic spectral parameters, too. Proline is a secondary amine, and after its involvement in a peptide bond, it cannot be deprotonated and cannot take part in metal binding [28,29]. As a consequence, amide coordination can occur only toward the C-terminus in the form of a seven-membered chelate. Its thermodynamic stability is much lower than those of the six- or five-membered ones and shifts the amide coordination to higher pH values. In the case of tau(404–409), (Ac-SPRHLS-NH_2_), where proline is the second amino acid on the N-terminal side of histidine, this affects only the formation of the 4N-coordinated complexes, resulting in a much higher p*K*_3amide_ value (see first column in Table 2 and Figure 2b). The effect of proline is even more pronounced if it directly precedes the histidyl residue. This is the case for tau(91–97) (Ac-AQPHTEI-NH_2_). The speciation curves for the copper(II)-tau(91–97) system are shown in Figure A3 in Appendix A. In this case, the species [CuL] predominates at around pH 7. 

On the one hand, it results from the stabilizing role of glutamyl carboxylate coordination, and on the other hand, it is caused by the suppression of amide coordination. It is also a common feature of the two peptides containing prolyl residues: at high pH, they form the species [CuH_−4_L], in which the imidazole-N donors are replaced by the fourth deprotonated amide nitrogen [30]. This change in coordinated donor groups can also be explained by the low thermodynamic stability of the seven-membered chelates.

In the Introduction, it has already been mentioned that, among the previously studied tau fragments, the heptapeptide tau(26–33) involving a His32 residue was the most effective copper(II)-binding ligand.

**Figure 3:**
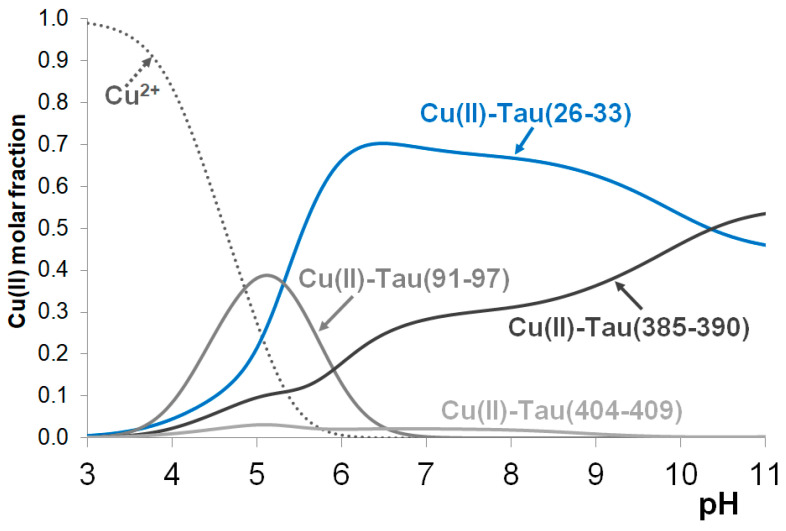
The concentration distribution of copper(II) ions in a model system containing copper(II) and the peptides tau(26–33), tau(91–97), tau(385–390) and tau(404–409) in equimolar concentrations (c(L) = 1 mM). Data from ref. [21].

Figure 3 shows the concentration distribution curves in a hypothetical system where copper(II), tau(26–33), tau(91–97), tau(385–390) and tau(404–409) are present in equimolar concentrations (1 mM). Speciation was calculated using the protonation constants of peptides and stability constants of complexes reported in this study and in ref. [21]. It is evident from Figure 3 that the predominance of the His32 binding site persists even in the presence of any of the studied peptides containing a single histidyl residue. 

### 2.3. Copper-Catalyzed Oxidation of Peptides

**Figure A4:**
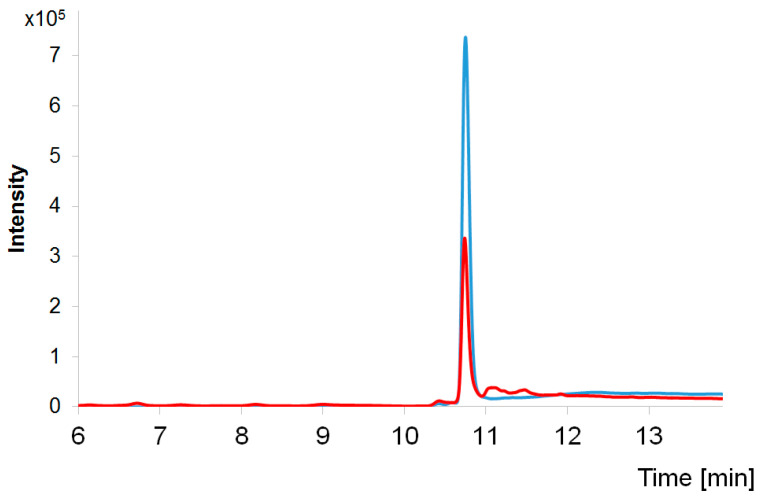
Representative HPLC of tau(385–390) (blue) and the Cu(II):tau(385–390):H_2_O_2_ = 1:1:4 system after 90 min of oxidation (red).

The oxidation of the peptides was studied with the Cu(II)/H_2_O_2_ system at pH 7.4 in the absence and presence of ascorbic acid. The coordination modes in the studied systems are quite different at this pH value. In the case of tau(91–97), Cu-N_im_-coordinated [CuL] is formed at the highest concentration (80%) besides the (N^−^,N^−^,N_im_)-coordinated [CuH_−2_L] species (see Figure A3 in Appendix A), while the latter is the main species with the other two studied peptides, forming at 94% and 98% besides the (N^−^,N^−^,N^−^,N_im_)-coordinated [CuH_−3_L] species with tau(385–390) and tau(404–409), respectively (Figure 1). However, the oxidation processes of these peptides are similar. In the absence of ascorbic acid, the fragmentation of the peptides occurs, and several products are formed. These reaction products either elute with a very small retention time or overlap with the peak of the non-oxidized ligand; therefore, the identification of these fragments with low molecular weights and/or very low intensities is not possible. However, in the presence of ascorbic acid, a new peak appears in the HPLC chromatogram of each system (Figure A4 in Appendix A). The retention time of this peak is always higher than the retention time of the peptide. 

**Figure 4:**
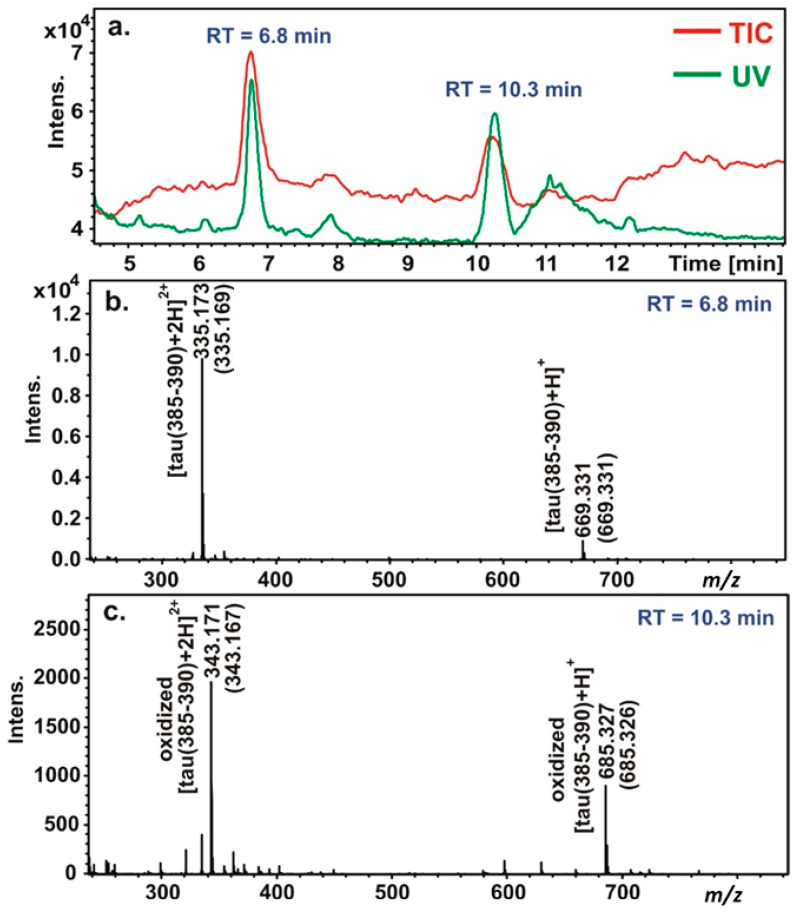
Representative LC-MS chromatogram of the Cu(II):tau(385–390):H_2_O_2_:ascorbic acid = 1:1:4:20 system. The total ion chromatogram (TIC) of MS and the UV chromatogram recorded at 222 nm (**a**); MS spectra obtained at RT = 6.8 min (**b**) and at RT = 10.3 min (**c**) (theoretical *m*/*z* values are in brackets).

The *m*/*z* values are higher with 16 and 8 Da in the case of ions with one and two positive charges, respectively. The main oxidation product was identified as the 2-oxo-histidine derivative of the peptides. A representative LC-MS chromatogram of tau(385–390) is presented in Figure 4.

The retention time (RT) of the peptide is 6.8 min. It appears both with only one and with two positive charges, whose *m*/*z* values are 669.331 (calculated value is 669.331) and 335.173 (calculated value is 335.169). The retention time of the oxidized product is 10.3 min. It appears with 685.327 and 343.171 *m*/*z* values (the calculated values are 685.326 and 343.167). It refers to the mono-oxidized product of the peptide, namely, the 2-oxo-derivative of the peptide. The higher retention time of the 2-oxo-derivative can be explained by the reduced basicity of the imidazole ring; the less basic compound elutes later with the acidic, TFA-containing eluent.

To sum up, we can conclude that the coordination mode of the formed complexes and the number of coordinating amide nitrogen atoms—if the coordination sphere of the Cu(II) ion is unsaturated—have no effect on the oxidation of the tau peptides. In the absence of ascorbic acid, the oxidative fragmentation of the tau peptides occurs; 40% of the peptides remain unchanged after 90 min oxidation.

In the presence of ascorbic acid, the extent of oxidation is slightly higher (about 30% of the peptides remain unchanged after 90 min oxidation), but the fragmentation of the peptide is less preferred, and the formation of 2-oxo-histidine occurs. Ascorbic acid has no protective role during the oxidation of tau peptides, in contrast to methionine-containing prion protein fragments [31].

### 2.4. Zinc(II) Complexes of Peptides

Zinc is one of the most important trace elements for almost all forms of life. It is the major constituent of a huge number of metalloenzymes and takes part in a series of biochemical processes. Many previous publications prove its involvement in neurodegeneration, but the exact role of zinc(II) ions in these disorders is not well understood. On the other hand, zinc(II) is an effective complex-forming ion with most of the various biomolecules containing either nitrogen, oxygen or sulfur donor atoms [29]. Zinc(II) complexes of peptides are also often studied, but the thermodynamic stability of these complexes is generally much lower than those of the corresponding copper(II) species [29]. The major difference in the copper(II) and zinc(II) peptide complexes is related to their affinity for amide binding. Copper(II) can easily promote the deprotonation and coordination of amide groups, forming stable five- or six-membered chelate rings, but in the case of zinc(II), this binding mode is rather rare and occurs only with specific sequences [29]. For example, peptide fragments of the prion protein have relatively low zinc(II)-binding affinity because the histidyl sites are well separated, and the number of other polar side chains is low [9]. On the contrary, amyloid-β can form stable complexes with both copper(II) and zinc(II) ions, and this can be explained by the presence of vicinal histidyl residues and a series of polar aspartic acid and glutamic acid side chains [8]. Recently, we reported the results obtained for the zinc(II) complexes of a series of tau peptide fragments including both the N-terminal and R3 regions of the protein [23]. This study revealed a relatively low zinc(II)-binding affinity of the His14 and His32 residues close to the N-terminus. On the contrary, the peptide and its mutants in the R3 region (tau(326–333)) containing the adjacent His329 and His330 residues formed rather stable zinc(II) complexes with the involvement of imidazole and deprotonated amide nitrogen donors in metal binding.

Potentiometric titrations of zinc(II)-containing systems revealed the very low zinc(II)-binding affinity of all three peptides (tau(91–97), tau(385–390) and tau(404–409)) involved in this study. In the case of tau(91–97) (Ac-AQPHTEI-NH_2_), the formation of a zinc(II)-hydroxide precipitate hindered the determination of any equilibrium data for any species, even in the presence of excess ligand. For tau(385–390) (Ac-KTDHGA-NH_2_), log *β*(ZnL) = 5.05(5) can be obtained, but taking into account the lysyl residue, which must be protonated in the acidic medium, the real stoichiometry of this species is [ZnH_−1_LH], containing an imidazole-coordinated and protonated ligand with a coordinated hydroxide ion. By subtracting the p*K* value of the lysyl ammonium group, log *β* = −5.29 can be calculated for the mixed hydroxide complex. Tau(404–409) has only one protonation site, and in this case, the stability constants for both the simple imidazole-N-coordinated and mixed hydroxido complexes can be directly obtained: log *β*([ZnL]) = 2.14 and log *β*([ZnH_−1_L]) = –4.68. These values are rather close to those obtained for other peptides containing a single histidyl residue but much lower than those of tau(9–16) (log *β*([ZnH_−1_L]) = −3.49) [23]. The latter peptide, however, contains two glutamic acids and one aspartic acid, and their carboxylate functions have a significant contribution to zinc(II) binding in addition to the imidazole-N donor atom. It is also important to note that the deprotonation and zinc(II) coordination of peptide amide groups were not observed for any ligands containing a single histidyl residue. The occurrence of this process was, however, reported for the tau(326–333) fragment [23] containing two vicinal histidyl sites. As a consequence, it can be unambiguously stated that the R3 region of tau protein is the most preferred zinc(II)-binding domain.

### 2.5. Nickel(II) Complexes of Peptides

Nickel(II) ions have a versatile coordination chemistry, and a great number of octahedral, square planar and tetrahedral complexes of this metal ion have already been characterized [32]. On the other hand, there are significant similarities in the complex formation processes of peptides with copper(II) and nickel(II) ions. Similar to copper(II), nickel(II) can promote the deprotonation and metal ion coordination of amide groups and form four-coordinated square planar complexes with tri- or longer peptides. The thermodynamic stability of the nickel(II)–peptide complexes is, however, lower than those of the corresponding copper(II) complexes, and these species are generally formed slightly above the physiological pH range. Our previous study on the nickel(II) complexes of peptides from the N-terminal and R3 domains of tau protein revealed that the complex formation processes of these peptides are quite similar to those of copper(II), and His32 was identified as the most preferred nickel(II) binding site [23]. These similarities suggest that nickel(II) can be effectively used as a probe to understand the complex formation processes of peptides with copper(II), although nickel(II) is not an essential element for human life.

The stability constants of the nickel(II) complexes of tau(91–97), tau(385–390) and tau(404–409) are listed in Table 4 in comparison with tau(9–16) and a mutant of tau(26–33). (The Gln/Lys mutation increased the aqueous solubility of the peptide without changing the coordination geometry [21,23]).

**Figure A5:**
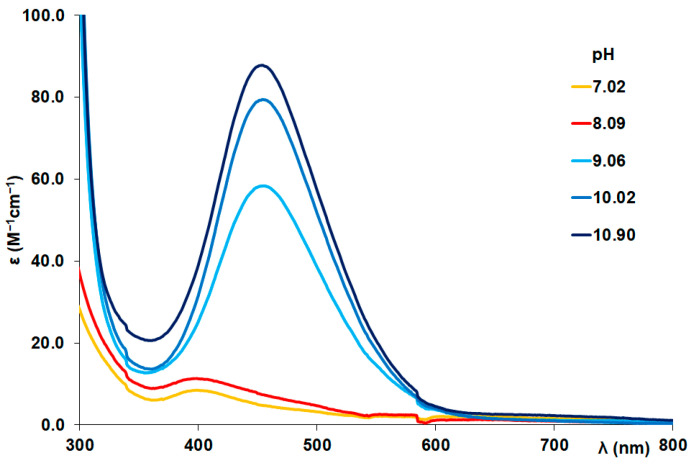
UV-Vis spectra registered in Ni(II)-tau(385–390) (Ac-KTDHGA-NH_2_) = 1:1 system (c(L) = 1.2 mM).

**Figure A6:**
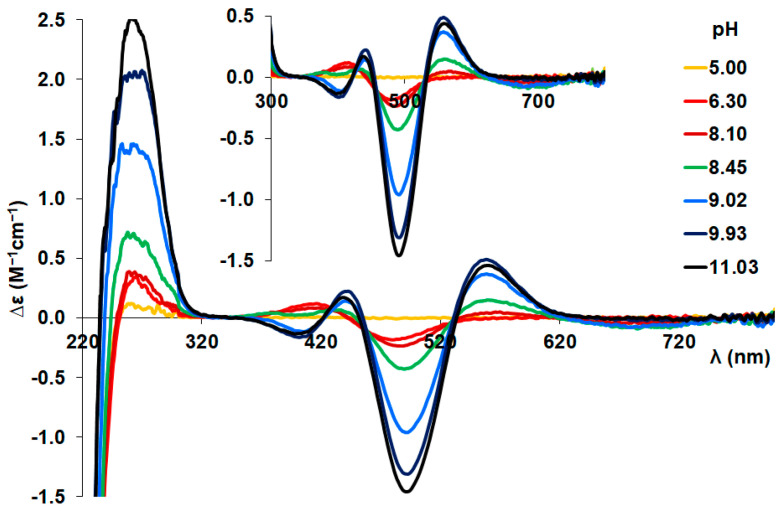
CD spectra registered in Ni(II)-tau(385–390) (Ac-KTDHGA-NH_2_) = 1:1 system (c(L) = 1.2 mM).

**Figure A7:**
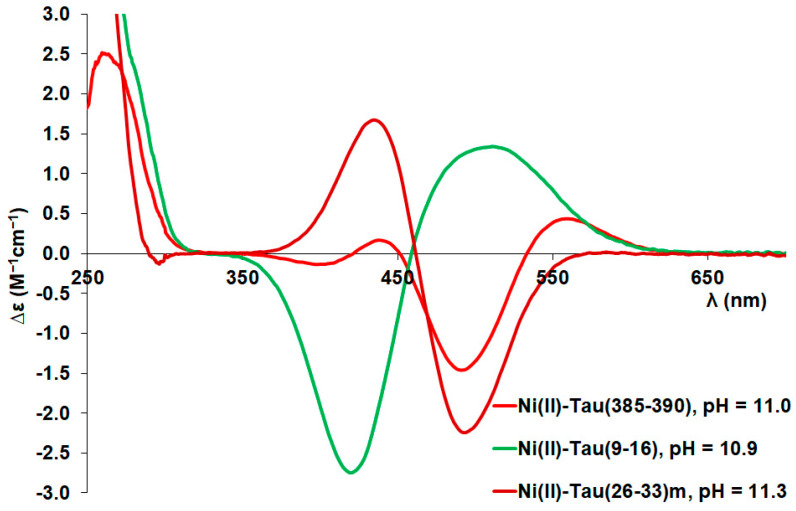
Visible circular dichroism (CD) spectra of (N^−^,N^−^,N^−^,N_im_)-coordinated [NiLH_−3_] (4N) complexes.

The evaluation of the data in Table 3 leads to some important conclusions:

1. The presence of proline significantly alters the complex formation processes of nickel(II) ions. This is especially true for tau(91–97), where amide coordination is not possible toward the N-terminus. In principle, it can occur on the C-terminal side of histidine, but the thermodynamic stability of the seven-membered chelate is much lower, and the corresponding species can be formed only under strongly alkaline conditions without any biological relevance. Thus, only the species [NiL] can be detected in the nickel(II)-tau(91–97) system. Its low stability constants, however, cannot prevent the hydrolysis of nickel(II) ions slightly above physiological pH.
2. In the nickel(II)-tau(404–409) system, the deprotonation and coordination of one amide group is possible, the (N_im_,N^−^) coordination mode, but the nickel(II) ion has two free coordination sites in this complex, resulting in hydrolytic reactions caused by increasing pH.
3. Complex formation processes in the nickel(II)-tau(385–390) and tau(26–33)(mutant) systems are quite similar to each other. The deprotonation of three amide groups is possible, resulting in the (N_im_,3N^−^) coordination mode above pH 8. The visible absorption (see Figure A5 in Appendix A) and circular dichroism spectroscopic measurements (see Figure A6 in Appendix A) strongly support the suggested coordination modes. It is also important to note that the pH-dependent changes in CD absorption are similar for the nickel(II) complexes of tau(385–390) and tau(26–33) mutant but differ from those of peptides missing the –TXH- sequence (Figure A7). A detailed investigation of the CD spectra of (N_im_,3N^−^)-coordinated Ni(II) complexes of peptides containing the -TXH- or -SXH- sequence [33,34] also shows that an intense negative extreme appears at around 500 nm, together with a positive peak between 430 and 451 nm. In some cases, an additional positive extremum can be observed at around 560 nm. The CD spectral pattern of the [NiH_−3_L] complex of tau(385–390) corresponds to the latter case [33].

**Figure 5:**
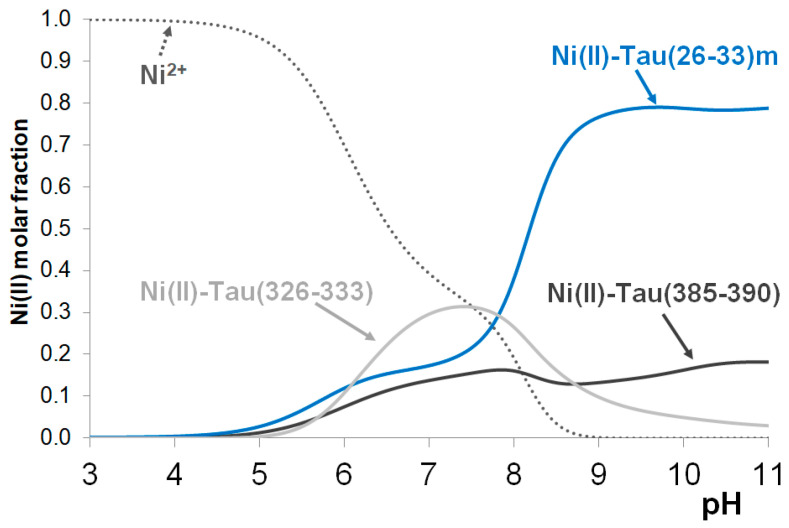
The concentration distribution of nickel(II) ions in a model system containing nickel(II) and the peptides tau(26–33)m, tau(326–333) and tau(385–390) in equimolar concentrations (c(L) = 1 mM). Data from refs. [21,23].

The major conclusions for the nickel(II) complexes, however, can be obtained from the hypothetical concentration distribution curves using the stability constants reported in this study and ref. [23] (Figure 5), where the distribution of nickel(II) ions between the three peptides tau(385–390), tau(26–33)m and tau(326–333) are plotted as a function of pH. It is evident from Figure 5 that the formation of stable nickel(II) complexes occurs only in slightly basic media (above pH~8) when both imidazole and amide nitrogen donors take part in metal binding. It is also obvious that (similar to copper(II) complexes) the fragment tau(26–33), close to the N-terminus, has the highest nickel(II)-binding affinity.

## 3. Materials and Methods

### 3.1. Materials

The peptides were purchased from SynPeptide Co., Ltd. (Shanghai, China) with purity levels above 95%. The concentrations of the peptide stock solutions were determined by pH-potentiometric titrations.

Stock solutions (CuCl_2_, NiCl_2,_ ZnCl_2_) were prepared from analytical-grade reagents (Reanal Zrt.), and their concentrations were verified gravimetrically via the precipitation of oxinate or using pH-potentiometric titrations with EDTA. KOH and KCl, used during pH-potentiometric titrations, were purchased from Merck (Waltham, MA, USA).

### 3.2. Potentiometric Measurements

pH-potentiometric titrations were completed on 3.00 mL samples at a 1–2 mM total ligand concentration with the aid of a carbonate-free stock solution of potassium hydroxide of known concentration. The metal-to-ligand ratios were selected between 1:3 and 1:1. During titration, argon was bubbled through the sample to ensure the absence of carbon dioxide. The samples were stirred by a VELP scientific magnetic stirrer. All pH-potentiometric measurements were carried out at 298 K and at a constant ionic strength of 0.2 M KCl. pH measurements were made with a MOLSPIN pH-meter equipped with a 6.0234.100 and 6.0234.110 combination glass electrode (Metrohm, Herisau, Switzerland), and the titrant was dispensed by means of a computer-controlled MOL-ACS burette. For the determination of the protonation constants of the ligands, two parallel titrations were usually performed, while for samples containing metal ions and peptides, several titration curves generated at different ratios ensured the reproducibility of the determined data. The number of experimental points was 50–100 for each titration curve. The recorded pH readings were converted into hydrogen ion concentrations, as described by Irving et al. [35]. The protonation constants of the ligands and the overall stability (log *β_pqr_*) constants of the metal complexes were calculated by means of general computational programs (SUPERQUAD [36] and PSEQUAD [37]) based on Equations (1) and (2). The uncertainties (*σ* values) in the stability constants are given in parentheses in the tables.

(1) $$p\;M+q\;H+r\;L⇌M_{p}H_{q}L_{r}$$

(2) $$\beta_{pqr}=\frac{{[M}_{p}H_{q}L_{r}]}{{\left[M\right]}^{p}{\left[H\right]}^{q}{\left[L\right]}^{r}}$$

The concentration distribution curves were generated by the MEDUSA program [38] using the protonation constants of the ligands and the stability constants of the metal ion complexes.

### 3.3. Spectroscopic Measurements (UV-Vis and CD Spectroscopy)

The same concentration range used for pH-potentiometry was selected to register the UV-Vis spectra of the copper(II) complexes. The measurements were carried out using a Perkin Elmer Lambda 25 scanning spectrophotometer in a wavelength range of 200–900 nm.

A JASCO J-810 spectropolarimeter (Jasco, Tokyo, Japan) was used to perform circular dichroism (CD) measurements. The CD spectra of the copper(II) complexes were acquired from 220 to 800 nm using 1 cm and 1 mm cells and at the same concentration as applied for pH-potentiometric measurements.

### 3.4. Oxidation of Tau Fragment

Reaction mixtures containing 1.0 mM peptide at a metal-to-ligand molar ratio of 1:1 were incubated at 25 °C for different time periods in the presence of hydrogen peroxide at a ligand-to-H_2_O_2_ molar ratio of 1:4 and in the simultaneous presence of hydrogen peroxide and ascorbic acid at a ligand-to-H_2_O_2_-to-ascorbic acid molar ratio of 1:4:20. The pH was adjusted to 7.4. The reaction was started by the addition of a 1% hydrogen peroxide solution, which was freshly prepared. After incubation, the reaction was stopped by the addition of Na_2_EDTA at a ligand-to-Na_2_EDTA ratio of 1:5. In the case of reaction mixtures containing ascorbic acid, the ligand-to-ascorbic acid molar ratio was also 1:20. The reaction process was monitored by RP-HPLC at different time periods.

### 3.5. Isolation of Oxidized Products

The samples were analyzed by analytical RP-HPLC using a Jasco instrument equipped with a Jasco MD-2010 plus multiwavelength detector. The oxidized products were analyzed on a Teknokroma Europa Protein 300 C8 (250 × 4.6 mm, 300 Å, 5 μm) at a flow rate of 1 mL·min^−1^, monitoring the absorbance at 222 nm. The mobile phases were water (A) and acetonitrile (B) containing 0.1% TFA. A linear gradient from 0 to 100% water containing 0.1% TFA was applied for 0 to 5 min. From 5 to 8 min, the mobile phase composition was changed to 80% water containing 0.1% TFA and 20% acetonitrile containing 0.1% TFA, and this was constant for 8 to 23 min. From 23 to 25 min, the mobile phase composition was changed to 100% water containing 0.1% TFA and remained the same until the end of the run. Each separation was stopped after 29.5 min.

### 3.6. HPLC-MS Measurements

HPLC-MS measurements were used to identify the products of the copper(II)-induced oxidation reactions and were performed by a MicroTOF-Q type Qq-TOF MS instrument (Bruker Daltonik, Bremen, Germany) operated in positive ion mode and a Waters 2695 Separations Module with a Teknokroma Europa Protein 300 C8 (250 × 4.6 mm, 300 Å, 5 μm), a thermostable autosampler (5 °C), a column module (35 °C) and a Waters 2996 Photodiode-array detector (PDA). The MS instrument was equipped with an electrospray ion source, where the spray voltage was 4 kV. N_2_ was utilized as a drying gas; the drying temperature was 200 °C, and the flow rate was 9.0 L/min. The same HPLC method was used as described above. The mass spectra were calibrated externally using the exact masses of clusters [(NaTFA)_n_^+^Na]^+^ generated from the electrosprayed solution of sodium trifluoroacetate (NaTFA). The spectra were evaluated with the DataAnalysis 3.4 software from Bruker. The analytes were detected with a PDA detector at λ = 222 nm, while the flow rate and the injection volume were 1.0 mL/min and 10 μL, respectively.

## 4. Conclusions

The studies reported in this manuscript unambiguously support the hypothesis that histidine-containing peptide fragments of tau protein outside the N-terminal domain and R1 and R3 regions can effectively bind metal ions, especially copper(II). The peptides tau(91–97) (Ac-AQPHTEI-NH_2_), tau(385–390) (Ac-KTDHGA-NH_2_) and tau(404–409) (Ac-SPRHLS-NH_2_) were involved in this study, and except for tau(385–390), the prolyl residue on the N-terminal side of histidine is also present in the sequence. Proline generally works as a break-point for amide coordination and leads to the lower thermodynamic stability of metal complexes. The reduced stability of complexes is especially true for nickel(II)- and zinc(II)-containing systems. Similar to other N-terminally protected peptides containing histidine, the imidazole-N donors of histidyl residues are the primary metal binding sites, but the deprotonated amide nitrogens can also take part in copper(II) and nickel(II) binding. Zinc(II)-promoted amide coordination has not been observed for any peptides, revealing that these domains of tau protein are not preferred zinc(II) binding sites. A comparison of the metal binding capabilities of these peptides with those of the N-terminal domain or the R1-R3 regions demonstrates that the His32 residue of tau protein has the highest copper(II) and nickel(II) binding affinity. The copper(II)-catalyzed oxidation of peptides has also been investigated in the presence of H_2_O_2_ and ascorbic acid. Both the fragmentation of peptides and the oxidation of histidyl residues to 2-oxohistidine have been detected, demonstrating that ascorbic acid has no protective role during the oxidation of tau peptides, in contrast to methionine-containing prion protein fragments.

## Figures and Tables

**Table 1:** Protonation constants of the peptides (T = 298 K, I = 0.2 mol/dm^3^ KCl).

Species	tau(91–97) (Ac-AQPHTEI-NH_2_)	tau(385–390) (Ac-KTDHGA-NH_2_)	tau(404–409) (Ac-SPRHLS-NH_2_)	tau(9–16) (Ac-EVMEDHAG-NH_2_) [21]	tau(26–33) (Ac-QGGYTMHQ-NH_2_) [21]
HL	6.60(1)	10.34(1)	6.24(1)	6.70	9.48
H_2_L	10.72(1)	16.85(1)	–	11.38	15.50
H_3_L	–	20.41(1)	–	15.52	–
H_4_L	–	–	–	18.93	–
p*K*(Im)	6.60	6.51	6.24	6.70	6.02
p*K*(Asp)	–	3.56	–	3.41	–
p*K*(Glu)_1_	4.12	–	–	4.14	–
p*K*(Glu)_2_	–	–	–	4.68	–
p*K*(Lys)	–	10.34	–	–	–
p*K*(Tyr)	–	–	–	–	9.48

**Table 2:** The stability constants (log *β_pqr_*) of the copper(II) complexes of the peptides (T = 298 K, I = 0.2 mol/dm^3^ KCl).

Species	tau(91–97) (Ac-AQPHTEI-NH_2_)	tau(385–390) (Ac-KTDHGA-NH_2_)	tau(404–409) (Ac-SPRHLS-NH_2_)	tau(9–16) (Ac-EVMEDHAG-NH_2_) [21]	tau(26–33) (Ac-QGGYTMHQ-NH_2_) [21]
[CuHL]	–	14.43(4)	–	9.66	13.26
[CuL]	5.02(4)	–	3.33(12)	5.04	–
[CuH_−1_L]	–	3.41(2)	–	–	3.23
[CuH_−2_L]	−10.73(7)	−5.24(4)	−8.22(4)	−8.09	−5.60
[CuH_−3_L]	–	−15.28(4)	−18.49(6)	−16.61	−15.47
[CuH_−4_L]	−30.20(7)	–	−29.18(4)	–	–
log*K*(Cu(II) + N_im_)	5.02	4.09	3.33	5.04	3.78
p*K*_1,2amide_ (av.)	7.875	5.51	5.775	6.57	5.01
p*K*_3amide_	9.735 (av.)	8.65	10.27	8.52	8.83
p*K*_4amide_		–	10.69	–	–

**Table 3:** UV-Vis parameters (λ_max_ [nm] (ε [M^−1^·cm^−1^]) of copper(II) complex fragments.

Coordination Mode	tau(91–97) (Ac-AQPHTEI-NH_2_)	tau(385–390) (Ac-KTDHGA-NH_2_)	tau(404–409) (Ac-SPRHLS-NH_2_)	tau(9–16) (Ac-EVMEDHAG-NH_2_) [21]	tau(26–33) (Ac-QGGYTMHQ-NH_2_) [21]
N_im_ (+COO^−^)	714(45)	750(15) *	790(15) *	747(38)	–
N^−^,N^−^,N_im_	622(98)	588(77)	602(84)	616(74)	590(75)
N^−^,N^−^,N^−^,N_im_	–	561(81)	577(85)	530(144)	551(87)
N^−^,N^−^,N^−^,N^−^	532(115)	–	531(174)	–	–

* The relatively low concentration of the complex makes the parameter uncertain.

**Table 4:** The stability constants (log *β_pqr_*) of the nickel(II) complexes of peptides (T = 298 K, I = 0.2 mol/dm^3^ KCl).

Species	tau(91–97) (Ac-AQPHTEI-NH_2_)	tau(385–390) (Ac-KTDHGA-NH_2_)	tau(404–409) (Ac-SPRHLS-NH_2_)	tau(9–16) (Ac-EVMEDHAG-NH_2_) [23]	tau(26–33)m (Ac-KGGYTMHK-NH_2_) [23]
[NiH_3_L]	–	–	–	–	32.92
[NiH_2_L]	–	–	–	–	25.29
[NiHL]	–	13.03(4)	–	–	–
[NiL]	4.20(6)	5.01(3)	1.98(9)	3.47	9.44
[NiH_–1_L]	−2.96(7)	–	−6.31(8)	−4.95	0.19
[NiH_–2_L]	–	−11.78(2)	–	–	–10.08
[NiH_–3_L]	–	−21.78(2)	–	−22.56	−20.52
log*K*(Ni(II) + N_im_)	4.20	2.69	1.98	3.47	2.70
p*K*_1amide_	–	8.09	8.29	8.42	7.67
p*K*_2,3amide_ (av)	–	8.40	–	8.805	7.92

## Data Availability

Data are contained within the article.
